# 2-(2,4-Dichloro­phen­yl)acetic acid

**DOI:** 10.1107/S1600536809052453

**Published:** 2009-12-12

**Authors:** Jiang-Sheng Li, Qi-Xi He, Peng-Yu Li

**Affiliations:** a School of Chemistry and Biological Engineering, Changsha University of Science & Technology, Changsha 410004, People’s Republic of China; b College of Chemistry and Chemical Engineering, Hunan University, Changsha 410082, People’s Republic of China

## Abstract

In the title compound, C_8_H_6_Cl_2_O_2_, the dihedral angle between the C—C(=O)—OH carboxyl unit and the benzene ring is 70.70 (4)°. In the crystal, mol­ecules are linked into inversion dimers by pairs of O—H⋯O hydrogen bonds. The dimers are linked into chains extending along [001] by weak C—H⋯Cl inter­actions.

## Related literature

For background to carboxylic acids as supra­molecular synthons, see: Thalladi *et al.* (1996 ▶). For related structures, see: Hodgson & Asplund (1991 ▶); Li *et al.* (2010 ▶).
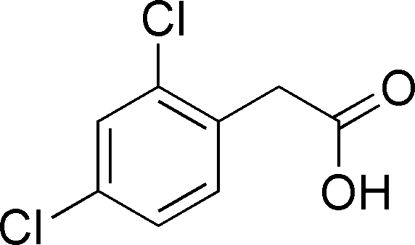


## Experimental

### 

#### Crystal data

C_8_H_6_Cl_2_O_2_
*M* *_r_* = 205.03
Monoclinic, 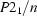

*a* = 10.824 (2) Å
*b* = 5.6061 (11) Å
*c* = 13.820 (3) Å
β = 91.08 (3)°
*V* = 838.4 (3) Å^3^
*Z* = 4
Mo *K*α radiation
μ = 0.72 mm^−1^
*T* = 113 K
0.24 × 0.20 × 0.12 mm

#### Data collection

Rigaku Saturn CCD diffractometer
Absorption correction: multi-scan (*CrystalClear*; Rigaku/MSC, 2005 ▶) *T*_min_ = 0.846, *T*_max_ = 0.918
5321 measured reflections
1484 independent reflections
1237 reflections with *I* > 2σ(*I*)
*R*_int_ = 0.037

#### Refinement

*R*[*F* ^2^ > 2σ(*F* ^2^)] = 0.027
*wR*(*F* ^2^) = 0.075
*S* = 1.10
1484 reflections
111 parameters
H-atom parameters constrained
Δρ_max_ = 0.23 e Å^−3^
Δρ_min_ = −0.22 e Å^−3^

### 

Data collection: *CrystalClear* (Rigaku/MSC, 2005 ▶); cell refinement: *CrystalClear*; data reduction: *CrystalClear*; program(s) used to solve structure: *SHELXS97* (Sheldrick, 2008 ▶); program(s) used to refine structure: *SHELXL97* (Sheldrick, 2008 ▶); molecular graphics: *SHELXTL* (Sheldrick, 2008 ▶); software used to prepare material for publication: *SHELXL97*.

## Supplementary Material

Crystal structure: contains datablocks I, global. DOI: 10.1107/S1600536809052453/hb5271sup1.cif

Structure factors: contains datablocks I. DOI: 10.1107/S1600536809052453/hb5271Isup2.hkl

Additional supplementary materials:  crystallographic information; 3D view; checkCIF report

## Figures and Tables

**Table 1:** Hydrogen-bond geometry (Å, °)

*D*—H⋯*A*	*D*—H	H⋯*A*	*D*⋯*A*	*D*—H⋯*A*
O2—H2⋯O1^i^	0.82	1.85	2.6689 (16)	175
C4—H4⋯Cl1^ii^	0.93	2.86	3.731 (2)	156

Symmetry codes: (i) 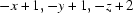; (ii) 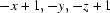.

**Fig. 1.**
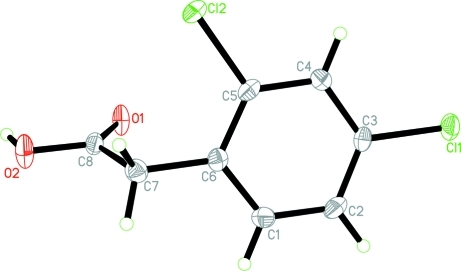
The molecular structure of (I) showing displacement ellipsoids drawn at the 50% probability level.

**Fig. 2.**
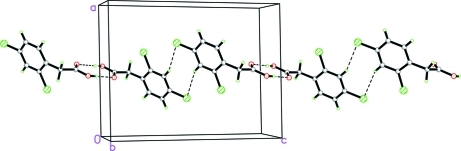
The infinite chain formed via alternative O—H···O and C—H···Cl hydrogen bonding down the c axis.

## supplementary crystallographic information

### Comment

Carboxylic acid is a supramolecular synthon, widely used to construct supramolecular array with one to three different dimensions *via* hydrogen bonds (Thalladi *et al.*, 1996). For our continuous research, we herein report the structure of the title compound (I).

In the title molecule, (Fig 1), the O1/O2/C7/C8 carboxyl unit forms an angle of 70.70 (4) A with the benzene ring. In the crystal packing, the molecules are linked into dimers by strong O—H···O H-bonding, which extend down the *c* axis by the aid of weak C—H···Cl H-bonding (Table 1 & Fig 2). For related structures, see: Hodgson & Asplund (1991) and Li *et al.* (2010).

### Experimental

The title compound was available from Hunan institute of Chemical Industry, received without further purification. Colourless blocks of (I) were obtained by evaporation from its solution of ethyl acetate/petroleum ether 1/2 (*v*/*v*).

### Refinement

All H atoms were positioned geometrically and constrained to ride on their parent atoms [C—H distances are 0.93 and 0.97Å with *U*_iso_(H) = 1.2 *U*_eq_(C) for aromatic and CH_2_ H atoms, 0.82Å with *U*_iso_ = 1.5*U*_eq_ (O) for hydroxyl H atom].

### Crystal data

**Table:**

C_8_H_6_Cl_2_O_2_	*F*(000) = 416
*M**_r_* = 205.03	*D*_x_ = 1.624 Mg m^−^^3^
Monoclinic, *P*2_1_/*n*	Melting point = 403–405 K
Hall symbol: -P 2yn	Mo *K*α radiation, λ = 0.71073 Å
*a* = 10.824 (2) Å	Cell parameters from 2684 reflections
*b* = 5.6061 (11) Å	θ = 2.4–27.9°
*c* = 13.820 (3) Å	µ = 0.72 mm^−^^1^
β = 91.08 (3)°	*T* = 113 K
*V* = 838.4 (3) Å^3^	Block, colourless
*Z* = 4	0.24 × 0.20 × 0.12 mm

### Data collection

**Table:**

Rigaku Saturn CCD diffractometer	1484 independent reflections
Radiation source: rotating anode	1237 reflections with *I* > 2σ(*I*)
confocal	*R*_int_ = 0.037
Detector resolution: 7.31 pixels mm^-1^	θ_max_ = 25.0°, θ_min_ = 2.4°
ω and φ scans	*h* = −12→12
Absorption correction: multi-scan (*CrystalClear*; Rigaku/MSC, 2005)	*k* = −6→6
*T*_min_ = 0.846, *T*_max_ = 0.918	*l* = −10→16
5321 measured reflections	

### Refinement

**Table:**

Refinement on *F*^2^	Secondary atom site location: difference Fourier map
Least-squares matrix: full	Hydrogen site location: inferred from neighbouring sites
*R*[*F*^2^ > 2σ(*F*^2^)] = 0.027	H-atom parameters constrained
*wR*(*F*^2^) = 0.075	*w* = 1/[σ^2^(*F*_o_^2^) + (0.0435*P*)^2^] where *P* = (*F*_o_^2^ + 2*F*_c_^2^)/3
*S* = 1.10	(Δ/σ)_max_ = 0.001
1484 reflections	Δρ_max_ = 0.23 e Å^−^^3^
111 parameters	Δρ_min_ = −0.22 e Å^−^^3^
0 restraints	Extinction correction: *SHELXL97* (Sheldrick, 2008), Fc^*^=kFc[1+0.001xFc^2^λ^3^/sin(2θ)]^-1/4^
Primary atom site location: structure-invariant direct methods	Extinction coefficient: 0.073 (5)

### Special details

**Table:**

Geometry. All e.s.d.'s (except the e.s.d. in the dihedral angle between two l.s. planes) are estimated using the full covariance matrix. The cell e.s.d.'s are taken into account individually in the estimation of e.s.d.'s in distances, angles and torsion angles; correlations between e.s.d.'s in cell parameters are only used when they are defined by crystal symmetry. An approximate (isotropic) treatment of cell e.s.d.'s is used for estimating e.s.d.'s involving l.s. planes.
Refinement. Refinement of *F*^2^ against ALL reflections. The weighted *R*-factor *wR* and goodness of fit *S* are based on *F*^2^, conventional *R*-factors *R* are based on *F*, with *F* set to zero for negative *F*^2^. The threshold expression of *F*^2^ > σ(*F*^2^) is used only for calculating *R*-factors(gt) *etc*. and is not relevant to the choice of reflections for refinement. *R*-factors based on *F*^2^ are statistically about twice as large as those based on *F*, and *R*- factors based on ALL data will be even larger.

### Fractional atomic coordinates and isotropic or equivalent isotropic displacement parameters (Å^2^)

**Table:**

	*x*	*y*	*z*	*U*_iso_*/*U*_eq_	
Cl1	0.71462 (4)	0.15511 (7)	0.45827 (3)	0.02309 (18)	
Cl2	0.35390 (4)	0.34376 (7)	0.70900 (3)	0.02349 (18)	
O1	0.55180 (10)	0.4443 (2)	0.88848 (8)	0.0230 (3)	
O2	0.46215 (12)	0.7778 (2)	0.94126 (8)	0.0266 (3)	
H2	0.4593	0.7022	0.9920	0.040*	
C1	0.67428 (16)	0.6663 (3)	0.65565 (12)	0.0196 (4)	
H1	0.7191	0.7977	0.6777	0.024*	
C2	0.72229 (14)	0.5277 (3)	0.58229 (11)	0.0203 (4)	
H2A	0.7977	0.5660	0.5551	0.024*	
C3	0.65595 (15)	0.3314 (3)	0.55033 (11)	0.0166 (4)	
C4	0.54302 (14)	0.2735 (3)	0.58966 (11)	0.0176 (4)	
H4	0.4990	0.1408	0.5680	0.021*	
C5	0.49744 (14)	0.4168 (3)	0.66162 (11)	0.0165 (4)	
C6	0.56134 (14)	0.6155 (3)	0.69727 (11)	0.0155 (4)	
C7	0.51115 (15)	0.7662 (3)	0.77720 (11)	0.0188 (4)	
H7A	0.5601	0.9107	0.7826	0.023*	
H7B	0.4272	0.8125	0.7601	0.023*	
C8	0.51103 (15)	0.6439 (3)	0.87375 (12)	0.0173 (4)	

### Atomic displacement parameters (Å^2^)

**Table:**

	*U*^11^	*U*^22^	*U*^33^	*U*^12^	*U*^13^	*U*^23^
Cl1	0.0220 (3)	0.0288 (3)	0.0187 (3)	0.00182 (16)	0.00584 (19)	−0.00592 (16)
Cl2	0.0160 (2)	0.0273 (3)	0.0274 (3)	−0.00391 (15)	0.00862 (19)	−0.00387 (17)
O1	0.0288 (6)	0.0256 (7)	0.0146 (6)	0.0115 (5)	0.0033 (5)	−0.0024 (5)
O2	0.0387 (8)	0.0245 (7)	0.0168 (6)	0.0114 (6)	0.0075 (6)	−0.0012 (5)
C1	0.0210 (9)	0.0183 (9)	0.0195 (9)	−0.0041 (6)	0.0008 (8)	0.0001 (7)
C2	0.0158 (8)	0.0250 (9)	0.0203 (9)	−0.0023 (7)	0.0046 (7)	0.0027 (7)
C3	0.0194 (8)	0.0192 (9)	0.0114 (8)	0.0035 (7)	0.0018 (7)	0.0015 (6)
C4	0.0179 (8)	0.0182 (8)	0.0168 (8)	−0.0013 (7)	−0.0004 (7)	−0.0024 (7)
C5	0.0130 (8)	0.0213 (8)	0.0153 (8)	−0.0004 (6)	0.0017 (6)	0.0047 (7)
C6	0.0193 (8)	0.0161 (8)	0.0111 (8)	0.0013 (6)	−0.0003 (7)	0.0028 (6)
C7	0.0210 (8)	0.0163 (8)	0.0191 (9)	0.0007 (7)	0.0004 (7)	−0.0005 (7)
C8	0.0135 (8)	0.0232 (10)	0.0151 (8)	0.0003 (6)	0.0017 (7)	−0.0044 (6)

### Geometric parameters (Å, °)

**Table:**

Cl1—C3	1.7405 (16)	C2—H2A	0.9300
Cl2—C5	1.7460 (16)	C3—C4	1.386 (2)
O1—C8	1.2185 (19)	C4—C5	1.377 (2)
O2—C8	1.316 (2)	C4—H4	0.9300
O2—H2	0.8200	C5—C6	1.396 (2)
C1—C2	1.386 (2)	C6—C7	1.501 (2)
C1—C6	1.390 (2)	C7—C8	1.500 (2)
C1—H1	0.9300	C7—H7A	0.9700
C2—C3	1.382 (2)	C7—H7B	0.9700
			
C8—O2—H2	109.5	C4—C5—Cl2	117.86 (12)
C2—C1—C6	122.16 (15)	C6—C5—Cl2	119.55 (13)
C2—C1—H1	118.9	C1—C6—C5	116.81 (15)
C6—C1—H1	118.9	C1—C6—C7	121.50 (14)
C3—C2—C1	118.72 (15)	C5—C6—C7	121.68 (15)
C3—C2—H2A	120.6	C8—C7—C6	113.81 (13)
C1—C2—H2A	120.6	C8—C7—H7A	108.8
C2—C3—C4	121.21 (15)	C6—C7—H7A	108.8
C2—C3—Cl1	119.43 (13)	C8—C7—H7B	108.8
C4—C3—Cl1	119.36 (12)	C6—C7—H7B	108.8
C5—C4—C3	118.50 (15)	H7A—C7—H7B	107.7
C5—C4—H4	120.7	O1—C8—O2	123.67 (16)
C3—C4—H4	120.7	O1—C8—C7	124.19 (15)
C4—C5—C6	122.59 (15)	O2—C8—C7	112.14 (13)
			
C6—C1—C2—C3	−0.6 (2)	C4—C5—C6—C1	0.9 (2)
C1—C2—C3—C4	0.5 (2)	Cl2—C5—C6—C1	−179.10 (11)
C1—C2—C3—Cl1	179.98 (12)	C4—C5—C6—C7	−178.68 (14)
C2—C3—C4—C5	0.2 (2)	Cl2—C5—C6—C7	1.3 (2)
Cl1—C3—C4—C5	−179.22 (11)	C1—C6—C7—C8	−109.95 (17)
C3—C4—C5—C6	−1.0 (2)	C5—C6—C7—C8	69.59 (19)
C3—C4—C5—Cl2	179.02 (11)	C6—C7—C8—O1	2.7 (2)
C2—C1—C6—C5	−0.1 (2)	C6—C7—C8—O2	−177.59 (13)
C2—C1—C6—C7	179.49 (14)		

### Hydrogen-bond geometry (Å, °)

**Table:**

*D*—H···*A*	*D*—H	H···*A*	*D*···*A*	*D*—H···*A*
O2—H2···O1^i^	0.82	1.85	2.6689 (16)	175
C4—H4···Cl1^ii^	0.93	2.86	3.731 (2)	156

Symmetry codes: (i) −*x*+1, −*y*+1, −*z*+2; (ii) −*x*+1, −*y*, −*z*+1.
